# Phenotypic Plasticity Determines Cancer Stem Cell Therapeutic Resistance in Oral Squamous Cell Carcinoma

**DOI:** 10.1016/j.ebiom.2016.01.007

**Published:** 2016-01-09

**Authors:** Adrian Biddle, Luke Gammon, Xiao Liang, Daniela Elena Costea, Ian C. Mackenzie

**Affiliations:** a Blizard Institute, Barts and The London School of Medicine and Dentistry, Queen Mary University of London, UK; b The Gade Laboratory of Pathology, Department of Clinical Medicine, University of Bergen, Norway

**Keywords:** Cancer, Stem cell, CSC, EMT, Plasticity, Therapy, Resistance

## Abstract

Cancer stem cells (CSCs) drive tumour spread and therapeutic resistance, and can undergo epithelial-to-mesenchymal transition (EMT) and mesenchymal-to-epithelial transition (MET) to switch between epithelial and post-EMT sub-populations. Examining oral squamous cell carcinoma (OSCC), we now show that increased phenotypic plasticity, the ability to undergo EMT/MET, underlies increased CSC therapeutic resistance within both the epithelial and post-EMT sub-populations. The post-EMT CSCs that possess plasticity exhibit particularly enhanced therapeutic resistance and are defined by a CD44^high^EpCAM^low/−^ CD24^+^ cell surface marker profile. Treatment with TGFβ and retinoic acid (RA) enabled enrichment of this sub-population for therapeutic testing, through which the endoplasmic reticulum (ER) stressor and autophagy inhibitor Thapsigargin was shown to selectively target these cells. Demonstration of the link between phenotypic plasticity and therapeutic resistance, and development of an *in vitro* method for enrichment of a highly resistant CSC sub-population, provides an opportunity for the development of improved chemotherapeutic agents that can eliminate CSCs.

## Highlights

• Cancer stem cell plasticity intersects with EMT to determine therapeutic resistance.
• Co-treatment with TGFβ and RA enriches a plastic and drug resistant cancer stem cell sub-population for therapeutic testing.
• Thapsigargin specifically targets the plastic and drug resistant cancer stem cell sub-population.

Cancer cells have the ability to switch between different identities that enhance their ability to proliferate and spread. In this report, we demonstrate that only some cancer cells possess this special ability to switch identity, and that these cells can resist current cancer therapies. We develop a method to enrich the cancer cells with this special ability, so that new drugs can be tested on them, and then use this method to identify a drug that can target these cells. These dangerous cells must be eradicated to enable long-term patient survival, and these are therefore significant findings for human health.

## 1 Introduction

Oral squamous cell carcinoma (OSCC) has an annual worldwide incidence of over 300,000 cases, a mortality rate of 48% (Torre et al., 2015), and commonly develops therapeutic resistance (da Silva et al., 2012). Resistance to therapeutic regimens is a major problem for cancer therapy, as it precludes complete ablation of the tumour and enables local and distant tumour recurrence, the main cause of cancer mortality. Cancer stem cells (CSCs), the sub-population of tumour cells that possess tumour-initiating potential (Clarke et al., 2006, Driessens et al., 2012), express heightened resistance to therapy compared to the majority non-stem cell population (Gupta et al., 2009, Li et al., 2008) and also drive tumour invasion and metastasis (Charafe-Jauffret et al., 2010, Hermann et al., 2007). In common with several other solid tumours, CSCs in OSCC are CD44^+^ (Prince et al., 2007). However it has recently become apparent that, despite being genetically homogenous, CSCs exhibit heterogeneous phenotypes (Biddle et al., 2011, Hermann et al., 2007, Liu et al., 2014). It has further been demonstrated that CSC heterogeneity within a tumour provides a non-genetic source of variation in therapeutic response (Kreso et al., 2013), although the CSC sub-populations underlying this variation have not been elucidated.

Epithelial-to-mesenchymal transition (EMT) is a developmental process in which epithelial cells acquire a migratory mesenchymal phenotype (Hay, 2005), and is often re-activated in cancer to drive tumour invasion and metastasis (Gjerdrum et al., 2010, Yang et al., 2008). In OSCC, the secreted cytokine TGFβ is an inducer of EMT and high EMT activity correlates with poor prognosis (Jensen et al., 2015). Mesenchymal-to-epithelial transition (MET), where migratory tumour cells revert back to an epithelial phenotype, also occurs in tumours and is often required in order to enable new tumour growth at metastatic sites (Tsai et al., 2012). Therefore, a level of phenotypic plasticity that enables sequential EMT and MET is important to tumour progression (Brabletz, 2012). This phenotypic plasticity is epigenetically regulated (Chaffer et al., 2013, Ke et al., 2010), and not all cells within the epithelial and post-EMT sub-populations possess the plasticity required to enable EMT/MET (Biddle et al., 2011, Chaffer et al., 2013).

We previously identified two distinct CSC sub-populations in OSCC: CD44^+^ EpCAM^high^ proliferative epithelial CSCs and CD44^high^EpCAM^low/−^ migratory/metastatic post-EMT CSCs (Biddle et al., 2011). Cells were able to switch between these two sub-populations by undergoing EMT and MET. Equivalent CSC sub-populations have now also been identified in breast cancer (Liu et al., 2014, Sarrio et al., 2012). We also identified a hierarchy of plasticity within the post-EMT CSC sub-population, such that only a subset of post-EMT CSCs could undergo MET to return to an epithelial phenotype (Biddle et al., 2011).

In the present study, we sought to examine the therapeutic resistance of heterogeneous CSC sub-populations in OSCC. We found that cellular phenotypic plasticity intersects with EMT to determine therapeutic resistance of CSCs. We identified a sub-population of post-EMT CSCs that are highly plastic, resistant to chemotherapeutic drugs including the CSC-targeted therapy (Gupta et al., 2009) salinomycin, and exhibit a CD44^high^EpCAM^low/−^ CD24^+^ cell surface marker profile. Their high plasticity and consequent re-establishment of heterogeneity posed a challenge for therapeutic testing, and we therefore developed a co-treatment regime of TGFβ with retinoic acid (RA) for stabilization and enrichment of this sub-population. Gene expression microarray analysis identified upregulation of processes involved in protein turnover in this sub-population, and this led us to identify Thapsigargin, a sarco/endoplasmic reticulum Ca^2 +^ ATPase (SERCA) inhibitor (Ganley et al., 2011), as a compound that selectively targets these cells. Finally, we demonstrated that fresh OSCC tumour specimens contain CSC sub-populations analogous to those identified in cell lines.

## 2 Materials and Methods

### 2.1 Cell Culture

Cell culture, including suspension culture for sphere assays, was performed as previously described (Biddle et al., 2011). The CA1 cell line was previously described (Biddle et al., 2011), and the LM cell line was recently derived in our laboratory from an OSCC of the mandibular region of the mouth. Cell removal from adherent culture was performed using 1 × Trypsin–EDTA (Sigma, T3924) at 37 °C. For TGFβ and RA treatment, cells were plated at a density of 10,000 cells per ml and TGFβ and RA were added to cell culture at the indicated concentrations. Medium and TGFβ and RA were replaced every two days for at least six days and until enough cells were produced for the required assays, whilst ensuring continued sub-confluence. Floating cells in culture are greatly enriched for the post-EMT sub-population; therefore, except for when treated with TGFβ or RA, the floating cells were used at each passage of pEMT-P in order to maintain the post-EMT sub-population.

### 2.2 Single Cell Cloning

Single cell cloning was performed as previously described (Biddle et al., 2011), using limiting dilution and microscopic examination of wells to identify those that contain a single cell. Clonal sub-lines were maintained under standard tissue culture conditions.

### 2.3 Flow Cytometry and FACS

Flow cytometry was performed as previously described (Biddle et al., 2011). Antibodies for cell line staining were CD44-PE (clone G44-26, BD Bioscience), CD24-FITC (clone ML5, BD Bioscience) and EpCAM-APC (clone HEA-125, Miltenyi Biotec). For fresh tumour cells, β4-integrin-PE (Epi-P39-9B, BD Bioscience) was added and CD44-PE was replaced with CD44-PerCP/Cy5.5 (clone G44-26, BD Bioscience). Single stained controls were performed for compensation, and isotype controls were performed to set negative gating.

### 2.4 Immunofluorescence

FACS sorted tumour cell sub-populations were smeared onto Poly-l-Lysine slides (Thermo Scientific) and incubated at 37 °C for 30 min to promote attachment. Cells were fixed in 4% paraformaldehyde, permeabilised with 0.25% Triton-X (Sigma) in phosphate buffered saline (PBS) (Sigma, D8662), and then blocked overnight in 1% bovine serum albumin (BSA) in PBS. Cells were then incubated overnight with primary antibodies in PBS/1% BSA, washed twice in PBS/1% BSA, and then incubated for 1 h with secondary antibodies in PBS/1% BSA. Cells were washed twice in PBS/1% BSA, incubated for 1 min with DAPI (Sigma) at 1 μg/ml in PBS, washed once in PBS and then mounted with Immu-Mount (Thermo Scientific). Imaging was performed at 200 × magnification, and exposure time was the same for all samples. Images were processed in Adobe Photoshop, with the threshold for high *versus* low staining the same for all samples. Antibody details can be found in the supplementary information.

### 2.5 RNA Extraction, cDNA Synthesis and QPCR

RNA extraction, cDNA synthesis and QPCR were performed as previously described (Biddle et al., 2011). Primer sequences are listed in the supplementary information.

### 2.6 Drug Dose Response Assays

Cells were plated at 1000 cells per well in flat-bottomed 96-well tissue culture plates (Corning). 24 h later, drugs were added at 4 different concentrations in triplicate technical replicates, with triplicate untreated control wells. 72 h after drug addition, cells were fixed in 4% paraformaldehyde and washed in PBS. For automated microscope analysis, cells were permeabilised with 0.1% Triton-X (Sigma) in PBS, then stained with CellMask deep red (Life Technologies H32721, used at 1:30,000 dilution) and 1 μg/ml DAPI (Sigma) for 1 h. Cells were washed twice with PBS. Cell images were acquired using an InCell 1000 automated microscope (GE), and then analysed using InCell Developer Toolbox software (GE) to determine the number of cells. Data was averaged for the triplicate technical replicates and normalized to the untreated wells. Results from at least three independent biological repeat experiments were entered into Graph-Pad Prism software to determine the dose response curve, IC50 and 95% confidence intervals for the IC50, using the nonlinear regression analysis of log(inhibitor) *versus* response with a variable slope. Drug details can be found in the supplementary information.

### 2.7 Microarray Analysis

RNA was extracted using the RNeasy microkit (Qiagen) and analysed using an Illumina Human HT-12 v4 gene expression array. The results were analysed using the GenomeStudio software (Illumina), with quantile normalization and a false discovery rate filter of 5% in differential expression analysis. The top 150 differentially expressed genes from each analysis were analysed with the functional annotation clustering tool on the DAVID database (Huang da et al., 2009a, Huang da et al., 2009b). Microarray data are deposited in the GEO database under the accession numbers GSE74578 and GSE74580.

### 2.8 Transplantation Into Immunodeficient Mice

NOD/SCID mice were obtained from Jackson Laboratories. Mice used in this study were of mixed gender and older than 6 weeks of age. The mice were maintained in a certified isolation facility under a pathogen free environment with standard 12/12 h day and night cycle, in accordance with European guidelines. All animal procedures were approved by the Norwegian Animal Research Authority. Cells were harvested from adherent culture and resuspended in 50 μl of Matrigel (BD Biosciences) on ice. The suspension was injected orthotopically into the tongues of NOD/SCID mice. Tumours were detected by palpation and the tumour volume was manually assessed with a digital calliper.

### 2.9 Isolation of Cells From Human Tumours

Tumour specimens were obtained from the pathology department at Barts Health NHS Trust, with full local ethical approval and patients' informed consent. Specimen site was selected to avoid both the tumour margin and necrotic core, and specimens were kept overnight at 4 °C in epithelial growth medium (termed FAD) with 10% FBS (Locke et al., 2005). Specimens were washed in PBS to remove blood, minced into approximately 1 mm^3^ pieces using scalpels, and then incubated with gentle agitation at 37 °C for 3 h with 2.5 mg/ml Collagenase type I (Sigma, C0130) in DMEM. An equal volume of DMEM containing 10% FBS was then added and the mixture was filtered through a 70 μm cell strainer prior to antibody staining for FACS.

### 2.10 Reagents

TGFβ (Millipore, GF111) was prepared as a 2 μg/ml stock solution in 4 mM HCl with 1 mg/ml BSA. Final concentrations were as indicated in the text. RA (Sigma, R2625) was prepared as a 10 mM stock solution in dimethyl sulfoxide (DMSO). Final concentration was 5 μM, a dose determined to be the highest dose that avoids toxicity.

### 2.11 Statistical Analysis

The number of biological repeats (n) for each experiment was at least three, except for the method development data in Fig. S2B–S2E and this is stated in the figure legend. Data is presented as mean ± s.e.m. Statistical analysis was performed using a two-sided t-test, and significance is indicated in the figures as * for P < 0.05 and ** for P < 0.01.

## 3 Results

### 3.1 CSC Plasticity Intersects With EMT to Determine Therapeutic Resistance

**Fig. 1:**
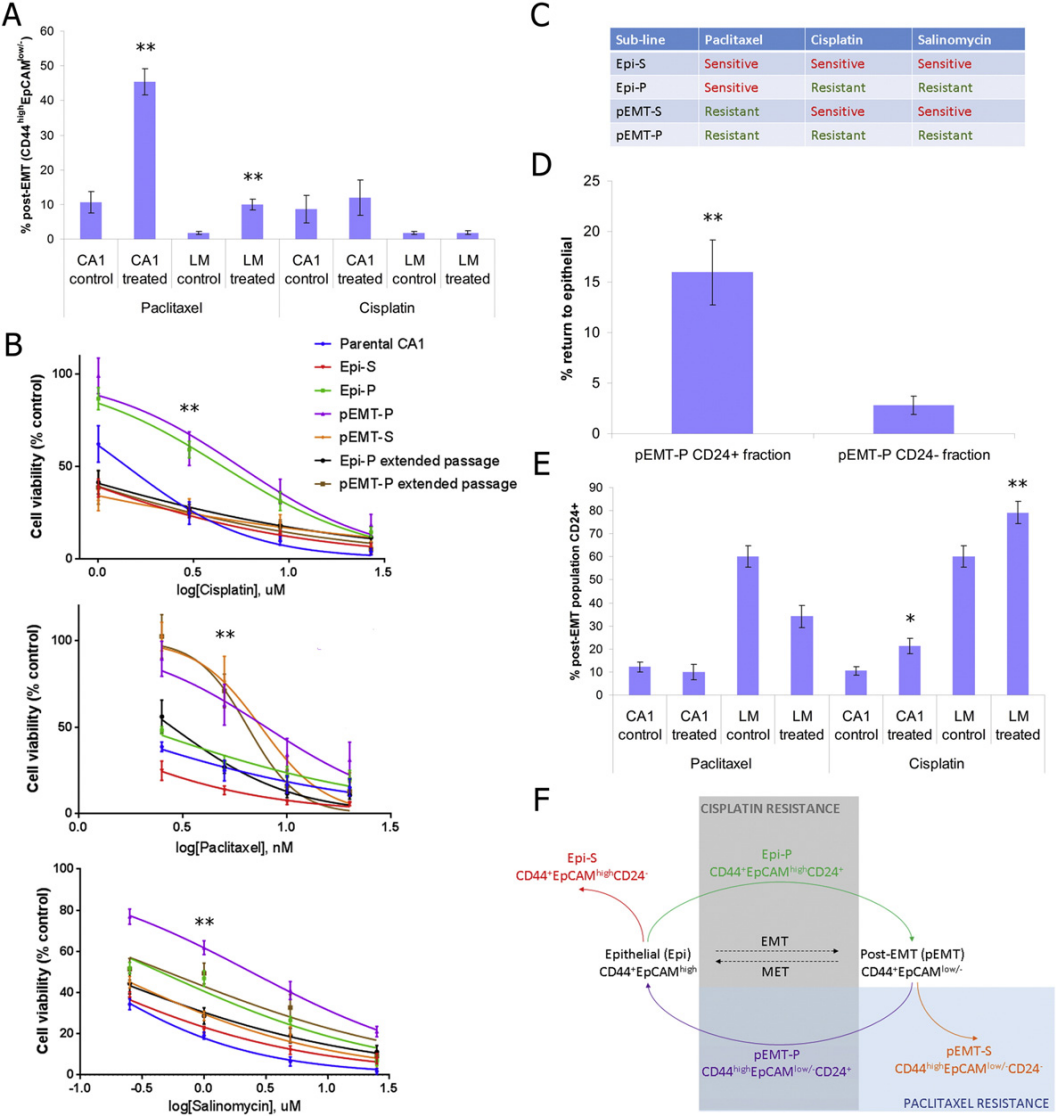
CSC plasticity intersects with EMT to determine therapeutic resistance. A, Flow cytometric analysis of the surviving cells after 3 days treatment of the CA1 and LM cell lines with 5 nM paclitaxel or 3 μM cisplatin followed by a 5 day recovery period. The size of the post-EMT sub-population (CD44^high^EpCAM^low/−^) as % of total cells (**P < 0.01). B, Dose response of each clonal sub-line and the parental CA1 cell line to cisplatin, paclitaxel and salinomycin. Cell viability expressed as number of cells remaining as a percentage of those in the control wells. Significance (**P < 0.01) was determined by comparison of Epi-P and pEMT-P to the parental CA1 line for cisplatin and salinomycin, and by comparison of pEMT-P to the parental CA1 line for paclitaxel. C, Summary of the results of the dose response assays, showing the sub-lines that exhibit resistance to each drug. D, The size of the epithelial sub-population (as % of total cells) in flow cytometric analysis 7 days after plating equal numbers of FACS sorted CD24^+^ and CD24^−^ cells from pEMT-P (**P < 0.01). E, Flow cytometric analysis of the surviving cells after 3 days treatment of the CA1 and LM cell lines with 5 nM paclitaxel or 3 μM cisplatin followed by a 5 day recovery period. Percentage of the post-EMT sub-population (CD44^high^EPCAM^low/−^) that is CD24^+^ (*P < 0.05, **P < 0.01). F, Scheme summarising the convergence of therapeutic resistance on a plastic post-EMT CSC sub-population, and indicating cell surface marker profiles for the four different CSC sub-populations (in colour). For the panels in this figure, n ≥ 3 biological repeats and error bars represent mean ± s.e.m. See also Fig. S1.

We initially examined the therapeutic resistance of post-EMT CSCs. We examined survival of CD44^high^EpCAM^low/−^ post-EMT CSCs in the CA1 and LM OSCC cell lines using two chemotherapeutic agents, paclitaxel and cisplatin (Fig. 1A). The post-EMT CSCs preferentially survived paclitaxel treatment, but showed no preferential survival of cisplatin treatment. This indicates that cisplatin resistance is not driven by EMT, and raises the question of whether resistance to cisplatin depends on alternative CSC sub-populations.

To investigate whether CSC plasticity could influence therapeutic response, we generated four clonal CA1 sub-lines which differ in their phenotypic plasticity: (1) a phenotypically stable epithelial sub-line with limited ability to undergo EMT (Epi-S); (2) a plastic epithelial sub-line with enhanced ability to undergo EMT (Epi-P); (3) a stable post-EMT sub-line unable to undergo MET (pEMT-S) (Biddle et al., 2011); and (4) a plastic post-EMT sub-line that can undergo MET (pEMT-P) (Biddle et al., 2011) (Fig. S1A and S1B). Despite maintenance of plasticity during initial derivation and early expansion, extended passage of both Epi-P and pEMT-P resulted in reduced plasticity and morphological changes (Fig. S1C and S1D), indicating a shift to a more Epi-S and pEMT-S-like state, respectively.

We investigated whether differences in plasticity could influence therapeutic response by assessing the responses of the four clonal sub-lines to the chemotherapeutic drugs paclitaxel and cisplatin (Figs. 1B and C and S1E). Salinomycin, an antibiotic previously reported to target post-EMT CSCs (Gupta et al., 2009), was also tested. Post-EMT sub-lines were resistant to paclitaxel, irrespective of plasticity, whereas epithelial sub-lines were sensitive. Contrastingly, plastic sub-lines (both Epi-P and pEMT-P) were resistant to both cisplatin and salinomycin, whereas stable sub-lines (both Epi-S and pEMT-S) were sensitive. pEMT-P, being both post-EMT and plastic, exhibited resistance to all three drugs. Extended passage resulted in loss of cisplatin and salinomycin resistance in both the Epi-P and pEMT-P sub-lines, whereas paclitaxel resistance was maintained over extended passage in the pEMT-P sub-line. This indicated that, whilst resistance to paclitaxel was determined by possession of a post-EMT identity, resistance to cisplatin and salinomycin was determined by possession of phenotypic plasticity and was lost as the clonal sub-lines lost their enhanced plasticity over extended passage.

We next sought to identify a cell surface marker that could be used to detect plastic CSCs in flow cytometry. CD24 was identified as a potential marker of plasticity from gene expression microarray analysis comparing Epi-P to Epi-S and pEMT-P to pEMT-S. The percentage of CD24^+^ cells was higher in flow cytometric analysis of Epi-P and pEMT-P compared to Epi-S and pEMT-S, respectively, and was reduced over extended passage (Fig. S1F and S1G) in concert with loss of plasticity and drug resistance. The CD24^+^ fraction from pEMT-P was enriched in the ability to undergo MET, relative to the CD24^−^ pEMT-P fraction (Fig. 1D), confirming CD24 as a marker of plasticity. To determine the contribution of the CD44^high^EpCAM^low/−^ CD24^+^ plastic post-EMT CSCs to therapeutic resistance, we examined their survival within the post-EMT sub-population in the CA1 and LM OSCC cell lines upon treatment with paclitaxel or cisplatin (Fig. 1E). The CD24^+^ plastic post-EMT CSCs preferentially survived cisplatin treatment, but showed no preferential survival of paclitaxel treatment compared to the rest of the post-EMT sub-population. This was in agreement with the findings above showing that post-EMT CSC sub-populations are resistant to paclitaxel irrespective of plasticity, whereas plasticity is important for resistance to cisplatin (Fig. 1F).

To determine if the four clonal sub-lines had different *in vivo* tumour initiating properties, we performed orthotopic xenograft studies in NOD/SCID mice. Injecting 5000 cells from Epi-S, Epi-P or pEMT-P produced tumours in 6/6, 5/6 and 5/6 mice respectively, whereas only 1 mouse out of 6 formed a tumour when injected with pEMT-S (Fig. S1H). Epi-S and the parental CA1 line both produced fast-growing tumours, Epi-P and pEMT-P produced slower-growing tumours, and the single tumour produced by pEMT-S only grew to a very small size (Fig. S1I). Importantly, pEMT-S produced the greatest number of tumourspheres in suspension culture (Fig. S1J), an assay of self-renewal that biases towards self-renewing post-EMT cells (Biddle et al., 2011, Chaffer et al., 2006). This supports the notion that the pEMT-S sub-population is not deficient in the ability to self-renew. Quantitative PCR with reverse transcription (QPCR) (Fig. S1K) showed that, compared to the parental CA1 line, pEMT-S and pEMT-P had upregulated expression of the EMT markers Vimentin and Zeb1 and downregulated expression of the epithelial markers E-cadherin and Keratin 15. The transcription factor Prrx1, which prevents MET (Ocana et al., 2012), was highly expressed in pEMT-S. Expression of Calgranulin B, a marker of epithelial terminal differentiation (Martinsson et al., 2005), was low in all four clonal sub-lines. These data demonstrate that both drug sensitive and drug resistant sub-populations contain self-renewing CSCs. There is evidence that post-EMT CSCs must undergo MET in order to drive new tumour formation (Ocana et al., 2012, Tsai et al., 2012), and our data suggest that the lack of plasticity of the pEMT-S sub-line underlies its inability to initiate tumour formation.

### 3.2 Co-treatment With TGFβ and RA Maintains Plasticity and Therapeutic Resistance

**Fig. 2:**
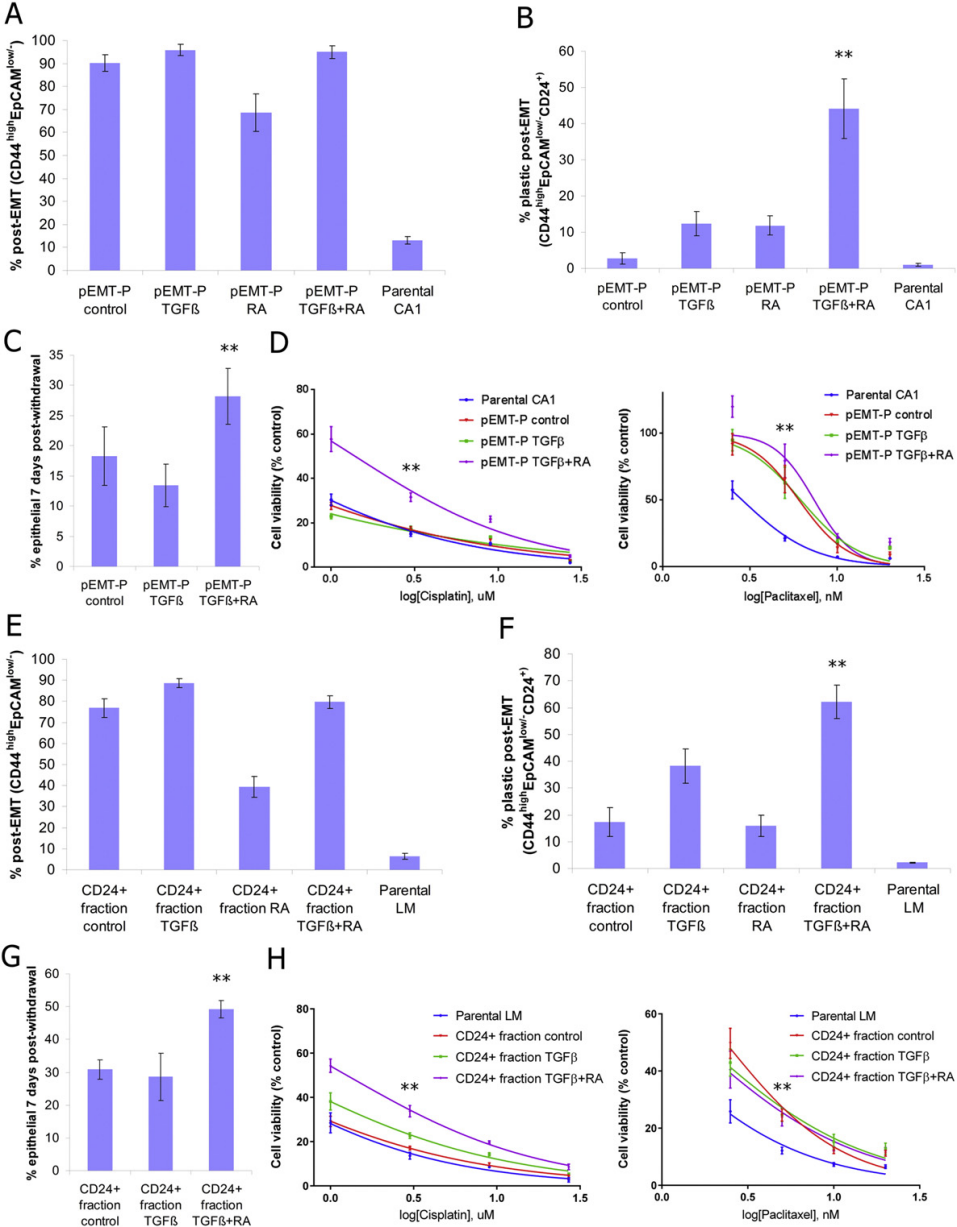
Maintaining plasticity also maintains therapeutic resistance. A and B, Flow cytometric analysis of pEMT-P untreated (control) or treated with TGFβ, RA, or TGFβ + RA, and the parental CA1 line. Size of the post-EMT sub-population (CD44^high^EpCAM^low/−^) (A), and size of the plastic post-EMT sub-population (CD44^high^EPCAM^low/−^ CD24^+^) (B), as % of total cells. C, Size of the epithelial sub-population (as % of total cells) 7 days after withdrawal of the different treatments from pEMT-P. D, Dose response to cisplatin and paclitaxel of pEMT-P after treatment with TGFβ and RA. Cell viability expressed as number of cells remaining as a percentage of those in the control wells. E and F, Flow cytometric analysis of the CD44^high^EpCAM^low/−^ CD24^+^ fraction (CD24 + fraction) from the LM line, untreated (control) or treated with TGFβ, RA, or TGFβ + RA, and the parental LM line. Size of the post-EMT sub-population (CD44^high^EpCAM^low/−^) (E), and size of the plastic post-EMT sub-population (CD44^high^EPCAM^low/−^ CD24^+^) (F), as % of total cells. G, Size of the epithelial sub-population (as % of total cells) 7 days after withdrawal of the different treatments from the LM CD44^high^EpCAM^low/−^ CD24^+^ fraction. H, Dose response to cisplatin and paclitaxel of the LM CD44^high^EpCAM^low/−^ CD24^+^ fraction after treatment with TGFβ and RA. Cell viability expressed as number of cells remaining as a percentage of those in the control wells. For the panels in this figure, n ≥ 3 biological repeats and error bars represent mean ± s.e.m. Significance (**P < 0.01) was determined by individual comparisons of TGFβ + RA treatment to each of the other treatments, except for the paclitaxel dose response curves where comparison was to the parental line only. See also Fig. S2.

We next sought to block the loss of plasticity that occurs over extended passage in pEMT-P, in order to determine whether this would also block the loss of cisplatin resistance. A method for experimental maintenance of cells in the plastic post-EMT sub-population would also be advantageous for therapeutic testing, as this sub-population exhibits resistance to all drugs tested so far. TGFβ induces EMT (Biddle et al., 2011), but extended TGFβ treatment drove the CA1 post-EMT sub-population into a stable post-EMT state (Fig. S2A). By contrast, RA induces epithelial differentiation (Metallo et al., 2008) and opposes TGFβ activity during development (Chen et al., 2007). We therefore tested whether RA could block the transition of pEMT-P into a pEMT-S-like state and act in concert with TGFβ to restrain cells in the plastic post-EMT sub-population. 5 μM RA caused increased MET of pEMT-P, creating a mixed population of epithelial and post-EMT cells, and had no effect on pEMT-S. The effect of RA was abrogated by 5 ng/ml TGFβ and did not prevent 5 ng/ml TGFβ from driving cells into a stable post-EMT state (Fig. S2B and S2C). However, by titrating the TGFβ dose we were able to establish a co-treatment regime that enabled maintenance of the plastic post-EMT sub-population (0.5 ng/ml TGFβ in combination with 5 μM RA) (Fig. S2D and S2E). Importantly, treatment of pEMT-P with 0.5 ng/ml TGFβ and 5 μM RA maintained this sub-line in the CD44^high^EpCAM^low/−^ CD24^+^ state (Figs. 2A and B and S2F and S2H). pEMT-P retained the ability to undergo MET upon treatment withdrawal (Fig. 2C), indicating maintenance of plasticity. Resistance to cisplatin and paclitaxel was maintained in pEMT-P treated with TGFβ and RA (Figs. 2D and S2I), demonstrating that maintained CSC plasticity results in maintenance of drug resistance.

To validate this protocol, we tested it using a second cell line. We FACS sorted the CD44^high^EpCAM^low/−^ CD24^+^ fraction from the LM OSCC cell line and treated the cells with TGFβ and RA during population expansion in culture. Treatment with TGFβ and RA maintained the cells in a CD44^high^EpCAM^low/−^ CD24^+^ state (Figs. 2E and F and S2G and S2H). The ability to undergo MET upon treatment withdrawal was also maintained (Fig. 2G), as was resistance to cisplatin and paclitaxel (Figs. 2H and S2J). Therefore, plasticity can be maintained in FACS sorted CD44^high^EpCAM^low/−^ CD24^+^ cells and this results in maintenance of drug resistance. In summary, we have developed a TGFβ + RA protocol for the enrichment of the plastic post-EMT CSC sub-population which dispenses with the need for single cell cloning experiments.

### 3.3 Thapsigargin Specifically Targets the Drug Resistant Plastic Post-EMT CSCs

**Fig. 3:**
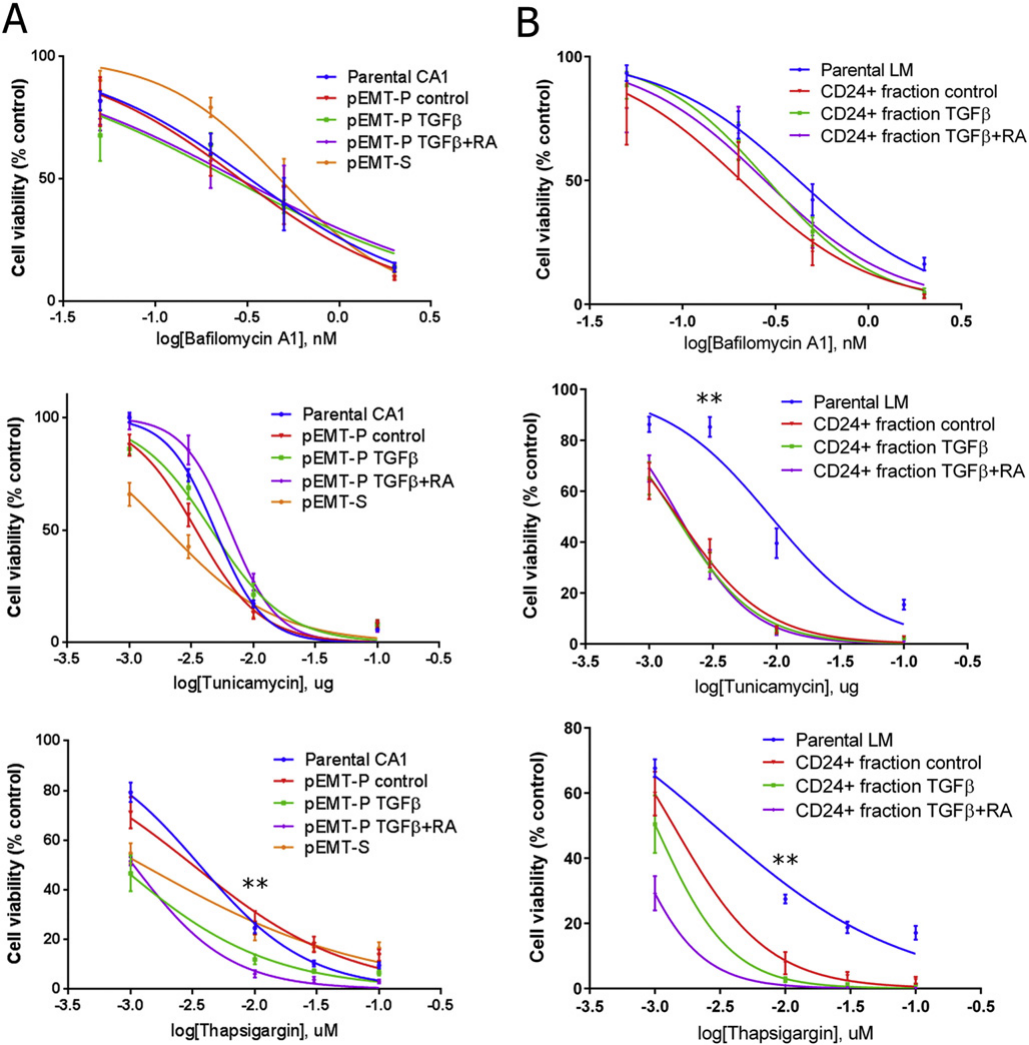
Thapsigargin specifically targets the drug resistant plastic post-EMT CSCs. A and B, Dose response to bafilomycin a1, tunicamycin and thapsigargin of pEMT-P (A) and the CD44^high^EpCAM^low/−^ CD24^+^ fraction (CD24 + fraction) from the LM line (B) after no treatment (control) or treatment with TGFβ or TGFβ + RA, and the corresponding parental line. Untreated pEMT-S was also included in A. Cell viability expressed as number of cells remaining as a percentage of those in the control wells. For the panels in this figure, n ≥ 3 biological repeats and error bars represent mean ± s.e.m. Significance (**P < 0.01) was determined by comparison of TGFβ + RA treatment to the corresponding parental line. See also Fig. S3.

To identify targetable cellular processes for therapeutic testing, we performed functional annotation clustering analysis of gene expression microarray data for genes upregulated in plastic sub-lines (Epi-P and pEMT-P compared to Epi-S and pEMT-S) and for genes upregulated in the TGFβ + RA protocol for retention of plasticity (TGFβ + RA treated pEMT-P and CD44^high^EpCAM^low/−^ CD24^+^ LM fraction compared to control and TGFβ treatments alone) (Table S1, Table S2, Table S3). Multiple cellular processes were upregulated in all three analyses, predominantly biosynthetic processes such as intracellular organelle production (including lysosomes and other membrane-bound organelles) and protein complex biogenesis, indicating high protein turnover. These processes may be required for the cellular remodelling intrinsic to phenotypic plasticity, and we attempted to sensitize the plastic post-EMT sub-population to cell death by targeting processes involved in protein turnover in TGFβ + RA treated pEMT-P and CD44^high^EpCAM^low/−^ CD24^+^ LM fraction. CX-5461, a selective inhibitor of RNApol1 (Drygin et al., 2011), did not cause increased death of the plastic post-EMT sub-population either alone or in combination with cisplatin (Fig. S3A–S3C). Bafilomycin A1, an inhibitor of lysosomal fusion (Yamamoto et al., 1998), and Tunicamycin, an inhibitor of protein folding in the endoplasmic reticulum (ER) that induces the ER unfolded protein response (UPR) (Xu et al., 2005), both induced increased death of post-EMT cells compared to the CA1 and LM parental lines (Figs. 3A and B and S3D and S3E). However, they did not specifically target the plastic post-EMT sub-population and targeting of post-EMT cells was weak. Thapsigargin, a SERCA inhibitor that both induces the ER UPR and inhibits lysosomal fusion (Ganley et al., 2011), specifically targeted the plastic post-EMT sub-population in both the CA1 and LM lines (Figs. 3A and B and S3D and S3E). Therefore, the TGFβ + RA protocol can be used to identify compounds that target the plastic post-EMT CSC sub-population, and we have identified Thapsigargin as one such compound.

### 3.4 OSCC Tumour Specimens Contain CSC Sub-populations With Corresponding Properties to Those Identified in Cell Lines

**Fig. 4:**
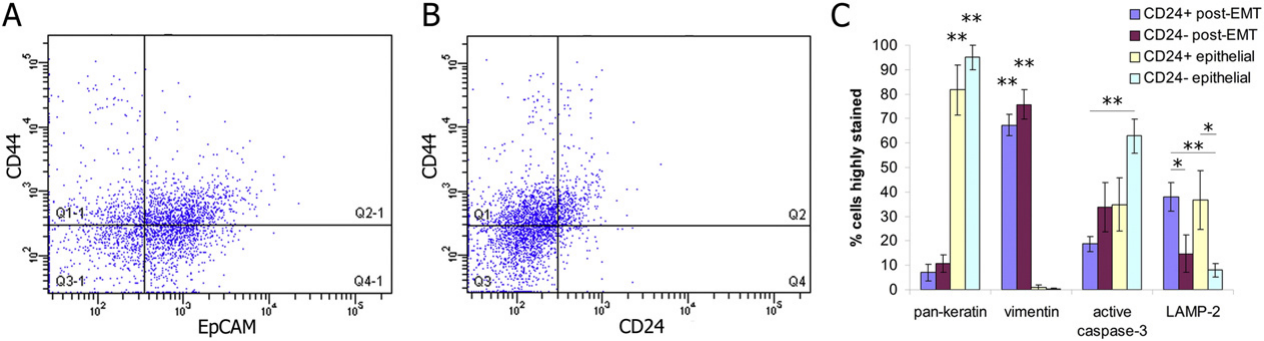
CSC sub-populations with the same properties as those identified *in vitro* also exist in fresh tumour specimens. A and B, Representative flow cytometric analysis of an OSCC tumour. CD44 and EpCAM distribution (y-axis; CD44, x-axis; EpCAM, crosshairs set with isotype control) (A), and CD44 and CD24 distribution (y-axis; CD44, x-axis; CD24, crosshairs set with isotype control) (B). Tumour cells were isolated from stromal cells using β4-integrin. C, Immunofluorescent antibody staining of FACS sorted cells prepared on slides. Percentage of cells from the four FACS sorted sub-populations that stain highly for pan-keratin, vimentin, active caspase-3 and LAMP-2. n = 7 tumours and error bars represent mean ± s.e.m. Significance (*P < 0.05, **P < 0.01) was determined by comparison of the epithelial fractions to the post-EMT fractions for pan-keratin and vimentin, and by comparisons indicated by horizontal lines for active caspase-3 and LAMP-2. See also Fig. S4.

We next investigated whether CSC sub-populations analogous to those identified in cell lines exist in fresh clinical specimens of OSCC. The distribution of CD44, EpCAM and CD24 staining on the specimen FACS plots corresponded with that seen in cell lines (Figs. 4A and B and S4A). Cells freshly isolated from OSCC tumours survive poorly *ex vivo*, so we assessed their attributes by direct antibody staining of FACS sorted post-EMT CD44^high^EpCAM^low/−^ CD24^+^ and CD44^high^EpCAM^low/−^ CD24^−^ fractions and epithelial CD44^+^ EpCAM^high^CD24^+^ and CD44^+^ EpCAM^high^CD24^−^ fractions from 7 specimens (Figs. 4C and S4B–S4H). Both the post-EMT CD44^high^EpCAM^low/−^ fractions stained highly for vimentin and low for pan-keratin, whereas both the epithelial CD44^+^ EpCAM^high^ fractions stained low for vimentin and highly for pan-keratin. This confirmed their post-EMT and epithelial identities, respectively. Activated caspase-3, a marker of cells entering apoptosis, was used to assess sensitivity to cell death. It was most highly expressed in the epithelial CD24^−^ fraction, had lowest expression in the post-EMT CD24^+^ fraction, and was expressed at an intermediate level in the epithelial CD24^+^ and post-EMT CD24^−^ fractions. This was in agreement with the therapeutic sensitivities of the corresponding cell line sub-populations. Lysosomal processes were important to plastic sub-populations in cell lines, so we stained tumour fractions for the lysosome marker LAMP-2. LAMP-2 was more highly expressed in the post-EMT CD24^+^ and epithelial CD24^+^ fractions than in either CD24^−^ fraction, again in agreement with the cell line data. These data indicate that CSC sub-populations exist in fresh tumour specimens with corresponding properties to those identified in cell lines.

## 4 Discussion

There has recently been considerable focus on the role of CSCs in tumour therapeutic resistance (Gupta et al., 2009, Li et al., 2008) but, despite the existence of heterogeneous CSC phenotypes (Biddle et al., 2011, Hermann et al., 2007, Liu et al., 2014), differences in therapeutic resistance between CSC sub-populations have not previously been investigated. We find that differing phenotypic plasticity is a key determinant of differences in therapeutic resistance between CSC sub-populations. Given the importance of phenotypic plasticity in tumour invasion and metastasis (Brabletz, 2012, Tsai et al., 2012), a sub-population of CSCs that exhibit both phenotypic plasticity and therapeutic resistance presents a potent threat and is likely to be an important target for therapeutic intervention. Our development of a method for enrichment of this most resistant CSC sub-population through co-treatment with TGFβ and RA provides a means for producing large numbers of these cells for drug development studies. This sub-population possesses a CD44^high^EpCAM^low/−^ CD24^+^ cell surface marker profile, and can therefore be monitored by flow cytometric analysis. As a proof of principle, we performed a targeted study and identified the SERCA inhibitor Thapsigargin as a compound to which this sub-population is selectively sensitive, demonstrating the potential of this method for therapeutic development. Identification of corresponding CSC sub-populations with corresponding resistance to cell death in fresh clinical specimens of OSCC indicates that this is an important new aspect of cancer biology with relevance to human tumours.

The precise mechanism linking plasticity with drug resistance remains to be determined. In this study, we identified the importance to plastic CSCs of processes involved in protein turnover. This suggests that rapid turnover of cellular contents might be required for cellular remodelling during transitions between phenotypic states, for which plastic CSCs are prepared. This rapid turnover is likely to exert considerable cellular stress, and the observed therapeutic resistance might be a by-product of mechanisms intended to protect the cell from stress during phenotypic transitions (Buchberger et al., 2010, Kroemer et al., 2010). We observed that plastic CSCs are extremely sensitive to Thapsigargin, which targets both lysosomal degradation and ER protein processing (Ganley et al., 2011). Interfering with ER protein processing would be expected to cause accumulation of misfolded proteins, and simultaneous inhibition of lysosomal degradation might prevent degradation of misfolded proteins so that they accumulate and overwhelm the cell. Plastic CSCs, undergoing rapid protein turnover, might be particularly sensitive to these perturbations.

In this study, CD24 was identified as a marker of plastic CSC sub-populations. CD24 is a mucin-like adhesion molecule expressed on the cell surface of multiple different cell types through a glycosylphosphatidylinositol membrane anchor, and high CD24 expression has been associated with more aggressive disease in ovarian, breast, lung and prostate cancer (Kristiansen et al., 2004). CD24 is a ligand for endothelial cell P-selectin, suggesting that it may act in metastatic dissemination of tumour cells (Aigner et al., 1997), and it has been shown to promote both tumour cell invasion (Bretz et al., 2012) and metastasis (Lau et al., 2014). CD24 was initially identified as a negative marker for CSCs in breast cancer (Al-Hajj et al., 2003), but has more recently been described as a positive CSC marker in lung cancer (Lau et al., 2014), colorectal cancer (Yeung et al., 2010), and triple-negative breast cancer (Azzam et al., 2013). It has been shown to mark a transient chemoresistant cell state in lung cancer (Sharma et al., 2010) and breast cancer (Goldman et al., 2015). Furthermore, CD24 has been implicated in regulation of EMT (Lim et al., 2014). Further studies into a potential role for CD24 in the control of CSC therapeutic resistance and plasticity may elucidate downstream regulatory mechanisms and suggest new therapeutic targets.

The analysis of fresh clinical specimens in this study provides a first indication that our *in vitro* model accurately models sub-populations existing in human tumours. However, to fully realise the potential of our *in vitro* model system for therapeutic development, future studies should further investigate its relevance to the *in vivo* disease state. Other tumour models, utilising fresh human tumour specimens, will need to be utilised to confirm the therapeutic responses of CSC sub-populations. It will also be important to investigate both a) the power of markers of plastic CSC sub-populations for predicting patient therapeutic response and b) the effect of clinical interventions in human subjects on markers of plastic CSC sub-populations.

This study has demonstrated that phenotypic plasticity underlies CSC therapeutic resistance. The demonstration that a highly plastic and drug resistant CSC sub-population can be stabilized by balancing opposing signalling pathways, and thus enriched *in vitro*, provides an opportunity for the development of novel therapeutic strategies targeting drug resistant CSCs that may otherwise survive therapeutic intervention and drive tumour recurrence. Given the similar underlying biology of epithelial tumours, these findings are likely to also be relevant to other cancers.

The following are the supplementary data related to this article.

Supplementary figures and methods.

Table S1 Functional annotation clustering for genes upregulated in pEMT-P and Epi-P.

Table S2 Functional annotation clustering for genes upregulated in TGFβ and RA treated pEMT-P.

Table S3 Functional annotation clustering for genes upregulated in TGFβ and RA treated LM CD24 + fraction.

## Conflict of Interest

The authors declare no conflicts of interest.

## Author Contributions

A.B. and I.C.M. designed the study and wrote the manuscript; A.B. performed the experiments; L.G. provided technical support and performed the automated microscope analysis; X.L. and D.E.C. performed the mouse transplantation experiments.

## References

[bb0005] Aigner S., Sthoeger Z.M., Fogel M., Weber E., Zarn J., Ruppert M., Zeller Y., Vestweber D., Stahel R., Sammar M., Altevogt P. (1997). CD24, a mucin-type glycoprotein, is a ligand for P-selectin on human tumor cells. Blood.

[bb0010] Al-Hajj M., Wicha M.S., Benito-Hernandez A., Morrison S.J., Clarke M.F. (2003). Prospective identification of tumorigenic breast cancer cells. Proc. Natl. Acad. Sci. U. S. A..

[bb0015] Azzam D.J., Zhao D., Sun J., Minn A.J., Ranganathan P., Drews-Elger K., Han X., Picon-Ruiz M., Gilbert C.A., Wander S.A. (2013). Triple negative breast cancer initiating cell subsets differ in functional and molecular characteristics and in gamma-secretase inhibitor drug responses. EMBO Mol. Med..

[bb0020] Biddle A., Liang X., Gammon L., Fazil B., Harper L.J., Emich H., Costea D.E., Mackenzie I.C. (2011). Cancer stem cells in squamous cell carcinoma switch between two distinct phenotypes that are preferentially migratory or proliferative. Cancer Res..

[bb0025] Brabletz T. (2012). EMT and MET in metastasis: where are the cancer stem cells?. Cancer Cell.

[bb0030] Bretz N., Noske A., Keller S., Erbe-Hofmann N., Schlange T., Salnikov A.V., Moldenhauer G., Kristiansen G., Altevogt P. (2012). CD24 promotes tumor cell invasion by suppressing tissue factor pathway inhibitor-2 (TFPI-2) in a c-Src-dependent fashion. Clin. Exp. Metastasis.

[bb0035] Buchberger A., Bukau B., Sommer T. (2010). Protein quality control in the cytosol and the endoplasmic reticulum: brothers in arms. Mol. Cell.

[bb0040] Chaffer C.L., Brennan J.P., Slavin J.L., Blick T., Thompson E.W., Williams E.D. (2006). Mesenchymal-to-epithelial transition facilitates bladder cancer metastasis: role of fibroblast growth factor receptor-2. Cancer Res..

[bb0045] Chaffer C.L., Marjanovic N.D., Lee T., Bell G., Kleer C.G., Reinhardt F., D'Alessio A.C., Young R.A., Weinberg R.A. (2013). Poised chromatin at the ZEB1 promoter enables breast cancer cell plasticity and enhances tumorigenicity. Cell.

[bb0050] Charafe-Jauffret E., Ginestier C., Iovino F., Tarpin C., Diebel M., Esterni B., Houvenaeghel G., Extra J.M., Bertucci F., Jacquemier J. (2010). Aldehyde dehydrogenase 1-positive cancer stem cells mediate metastasis and poor clinical outcome in inflammatory breast cancer. Clin. Cancer Res..

[bb0055] Chen F., Desai T.J., Qian J., Niederreither K., Lu J., Cardoso W.V. (2007). Inhibition of Tgf beta signaling by endogenous retinoic acid is essential for primary lung bud induction. Development.

[bb0060] Clarke M.F., Dick J.E., Dirks P.B., Eaves C.J., Jamieson C.H., Jones D.L., Visvader J., Weissman I.L., Wahl G.M. (2006). Cancer stem cells—perspectives on current status and future directions: AACR Workshop on cancer stem cells. Cancer Res..

[bb0065] da Silva S.D., Hier M., Mlynarek A., Kowalski L.P., Alaoui-Jamali M.A. (2012). Recurrent oral cancer: current and emerging therapeutic approaches. Front. Pharmacol..

[bb0070] Driessens G., Beck B., Caauwe A., Simons B.D., Blanpain C. (2012). Defining the mode of tumour growth by clonal analysis. Nature.

[bb0075] Drygin D., Lin A., Bliesath J., Ho C.B., O'Brien S.E., Proffitt C., Omori M., Haddach M., Schwaebe M.K., Siddiqui-Jain A. (2011). Targeting RNA polymerase I with an oral small molecule CX-5461 inhibits ribosomal RNA synthesis and solid tumor growth. Cancer Res..

[bb0080] Ganley I.G., Wong P.M., Gammoh N., Jiang X. (2011). Distinct autophagosomal–lysosomal fusion mechanism revealed by thapsigargin-induced autophagy arrest. Mol. Cell.

[bb0085] Gjerdrum C., Tiron C., Hoiby T., Stefansson I., Haugen H., Sandal T., Collett K., Li S., McCormack E., Gjertsen B.T. (2010). Axl is an essential epithelial-to-mesenchymal transition-induced regulator of breast cancer metastasis and patient survival. Proc. Natl. Acad. Sci. U. S. A..

[bb0090] Goldman A., Majumder B., Dhawan A., Ravi S., Goldman D., Kohandel M., Majumder P.K., Sengupta S. (2015). Temporally sequenced anticancer drugs overcome adaptive resistance by targeting a vulnerable chemotherapy-induced phenotypic transition. Nat. Commun..

[bb0095] Gupta P.B., Onder T.T., Jiang G., Tao K., Kuperwasser C., Weinberg R.A., Lander E.S. (2009). Identification of selective inhibitors of cancer stem cells by high-throughput screening. Cell.

[bb0100] Hay E.D. (2005). The mesenchymal cell, its role in the embryo, and the remarkable signaling mechanisms that create it. Dev. Dyn..

[bb0105] Hermann P.C., Huber S.L., Herrler T., Aicher A., Ellwart J.W., Guba M., Bruns C.J., Heeschen C. (2007). Distinct populations of cancer stem cells determine tumor growth and metastatic activity in human pancreatic cancer. Cell Stem Cell.

[bb0110] Huang da W., Sherman B.T., Lempicki R.A. (2009). Bioinformatics enrichment tools: paths toward the comprehensive functional analysis of large gene lists. Nucleic Acids Res..

[bb0115] Huang da W., Sherman B.T., Lempicki R.A. (2009). Systematic and integrative analysis of large gene lists using DAVID bioinformatics resources. Nat. Protoc..

[bb0120] Jensen D.H., Dabelsteen E., Specht L., Fiehn A.M., Therkildsen M.H., Jonson L., Vikesaa J., Nielsen F.C., von Buchwald C. (2015). Molecular profiling of tumour budding implicates TGF-beta-mediated epithelial-mesenchymal transition as a therapeutic target in oral squamous cell carcinoma. J. Pathol..

[bb0125] Ke X.S., Qu Y., Cheng Y., Li W.C., Rotter V., Oyan A.M., Kalland K.H. (2010). Global profiling of histone and DNA methylation reveals epigenetic-based regulation of gene expression during epithelial to mesenchymal transition in prostate cells. BMC Genomics.

[bb0130] Kreso A., O'Brien C.A., van Galen P., Gan O.I., Notta F., Brown A.M., Ng K., Ma J., Wienholds E., Dunant C. (2013). Variable clonal repopulation dynamics influence chemotherapy response in colorectal cancer. Science.

[bb0135] Kristiansen G., Sammar M., Altevogt P. (2004). Tumour biological aspects of CD24, a mucin-like adhesion molecule. J. Mol. Histol..

[bb0140] Kroemer G., Marino G., Levine B. (2010). Autophagy and the integrated stress response. Mol. Cell.

[bb0145] Lau A.N., Curtis S.J., Fillmore C.M., Rowbotham S.P., Mohseni M., Wagner D.E., Beede A.M., Montoro D.T., Sinkevicius K.W., Walton Z.E. (2014). Tumor-propagating cells and Yap/Taz activity contribute to lung tumor progression and metastasis. EMBO J..

[bb0150] Li X., Lewis M.T., Huang J., Gutierrez C., Osborne C.K., Wu M.F., Hilsenbeck S.G., Pavlick A., Zhang X., Chamness G.C. (2008). Intrinsic resistance of tumorigenic breast cancer cells to chemotherapy. J. Natl. Cancer Inst..

[bb0155] Lim J., Lee K.M., Shim J., Shin I. (2014). CD24 regulates stemness and the epithelial to mesenchymal transition through modulation of Notch1 mRNA stability by p38MAPK. Arch. Biochem. Biophys..

[bb0160] Liu S., Cong Y., Wang D., Sun Y., Deng L., Liu Y., Martin-Trevino R., Shang L., McDermott S.P., Landis M.D. (2014). Breast cancer stem cells transition between epithelial and mesenchymal states reflective of their normal counterparts. Stem Cell Rep..

[bb0165] Locke M., Heywood M., Fawell S., Mackenzie I.C. (2005). Retention of intrinsic stem cell hierarchies in carcinoma-derived cell lines. Cancer Res..

[bb0170] Martinsson H., Yhr M., Enerback C. (2005). Expression patterns of S100A7 (psoriasin) and S100A9 (calgranulin-B) in keratinocyte differentiation. Exp. Dermatol..

[bb0175] Metallo C.M., Ji L., de Pablo J.J., Palecek S.P. (2008). Retinoic acid and bone morphogenetic protein signaling synergize to efficiently direct epithelial differentiation of human embryonic stem cells. Stem Cells.

[bb0180] Ocana O.H., Corcoles R., Fabra A., Moreno-Bueno G., Acloque H., Vega S., Barrallo-Gimeno A., Cano A., Nieto M.A. (2012). Metastatic colonization requires the repression of the epithelial–mesenchymal transition inducer Prrx1. Cancer Cell.

[bb0185] Prince M.E., Sivanandan R., Kaczorowski A., Wolf G.T., Kaplan M.J., Dalerba P., Weissman I.L., Clarke M.F., Ailles L.E. (2007). Identification of a subpopulation of cells with cancer stem cell properties in head and neck squamous cell carcinoma. Proc. Natl. Acad. Sci. U. S. A..

[bb0190] Sarrio D., Franklin C.K., Mackay A., Reis-Filho J.S., Isacke C.M. (2012). Epithelial and mesenchymal subpopulations within normal basal breast cell lines exhibit distinct stem cell/progenitor properties. Stem Cells.

[bb0195] Sharma S.V., Lee D.Y., Li B., Quinlan M.P., Takahashi F., Maheswaran S., McDermott U., Azizian N., Zou L., Fischbach M.A. (2010). A chromatin-mediated reversible drug-tolerant state in cancer cell subpopulations. Cell.

[bb0200] Torre L.A., Bray F., Siegel R.L., Ferlay J., Lortet-Tieulent J., Jemal A. (2015). Global cancer statistics, 2012. CA Cancer J. Clin..

[bb0205] Tsai J.H., Donaher J.L., Murphy D.A., Chau S., Yang J. (2012). Spatiotemporal regulation of epithelial–mesenchymal transition is essential for squamous cell carcinoma metastasis. Cancer Cell.

[bb0210] Xu C., Bailly-Maitre B., Reed J.C. (2005). Endoplasmic reticulum stress: cell life and death decisions. J. Clin. Invest..

[bb0215] Yamamoto A., Tagawa Y., Yoshimori T., Moriyama Y., Masaki R., Tashiro Y. (1998). Bafilomycin A1 prevents maturation of autophagic vacuoles by inhibiting fusion between autophagosomes and lysosomes in rat hepatoma cell line, H-4-II-E cells. Cell Struct. Funct..

[bb0220] Yang M.H., Wu M.Z., Chiou S.H., Chen P.M., Chang S.Y., Liu C.J., Teng S.C., Wu K.J. (2008). Direct regulation of TWIST by HIF-1alpha promotes metastasis. Nat. Cell Biol..

[bb0225] Yeung T.M., Gandhi S.C., Wilding J.L., Muschel R., Bodmer W.F. (2010). Cancer stem cells from colorectal cancer-derived cell lines. Proc. Natl. Acad. Sci. U. S. A..

