# Supplementary material for: Phenotypic Plasticity Determines Cancer Stem Cell Therapeutic Resistance in Oral Squamous Cell Carcinoma
Source: eBioMedicine. 2016 Jan 9;4:138–45. doi: 10.1016/j.ebiom.2016.01.007 (PMC4776071; doi:10.1016/j.ebiom.2016.01.007)
Supplement: Supplementary file 1 — Supplementary figures and methods. [file mmc1.pdf]

Figure S1

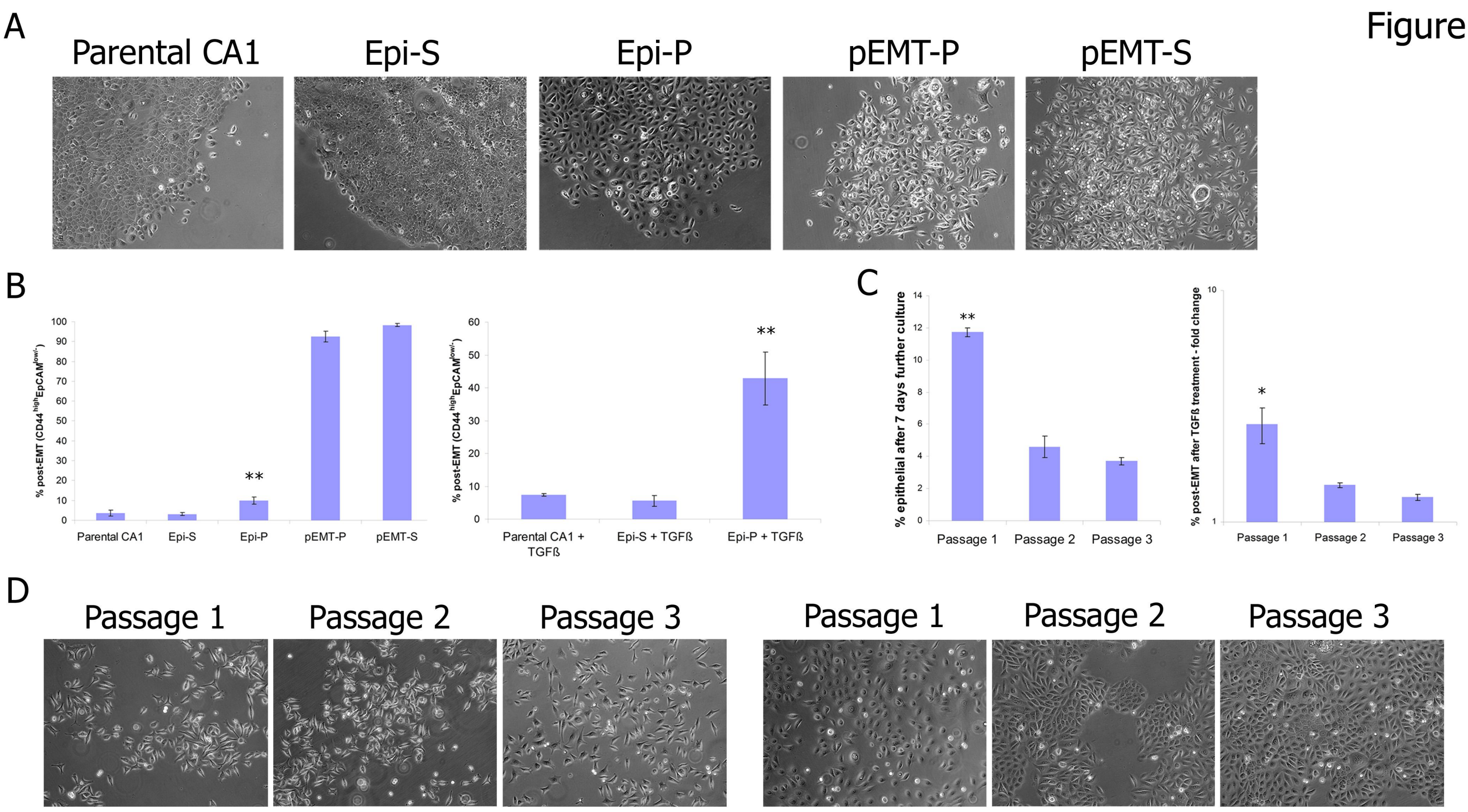

E

	Parental CA1	Epi-S	Epi-P	pEMT-P	pEMT-S	Epi-P extended passage	pEMT-P extended passage
Cisplatin IC50	1.412	0.51	4.496	5.529	0.2059	0.4946	0.4777
Cisplatin IC50 95% confidence intervals	1.043 to 1.911	0.2908 to 0.8945	3.517 to 5.748	3.639 to 8.402	0.03479 to 1.219	0.2316 to 1.056	0.1756 to 1.300
Paclitaxel IC50	1.175	0.786	1.922	7.926	7.497	2.807	6.479
Paclitaxel IC50 95% confidence intervals	0.4636 to 2.976	0.3386 to 1.824	1.058 to 3.490	5.179 to 12.13	5.825 to 9.648	2.157 to 3.653	5.272 to 7.961
Salinomycin IC50	0.09444	0.07629	0.4465	2.414	0.1669	0.1302	0.5052
Salinomycin IC50 95% confidence intervals	0.06059 to 0.1472	0.03963 to 0.1469	0.2792 to 0.7139	1.766 to 3.298	0.09354 to 0.2977	0.05327 to 0.3182	0.2072 to 1.232

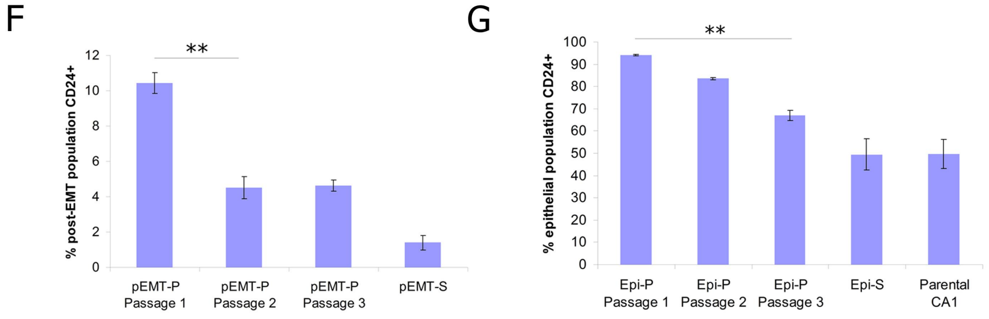

H

	Tumour incidence from 5000 cells
Parental CA1	6 of 6
Epi-S	6 of 6
Epi-P	5 of 6
pEMT-P	5 of 6
pEMT-S	1 of 6

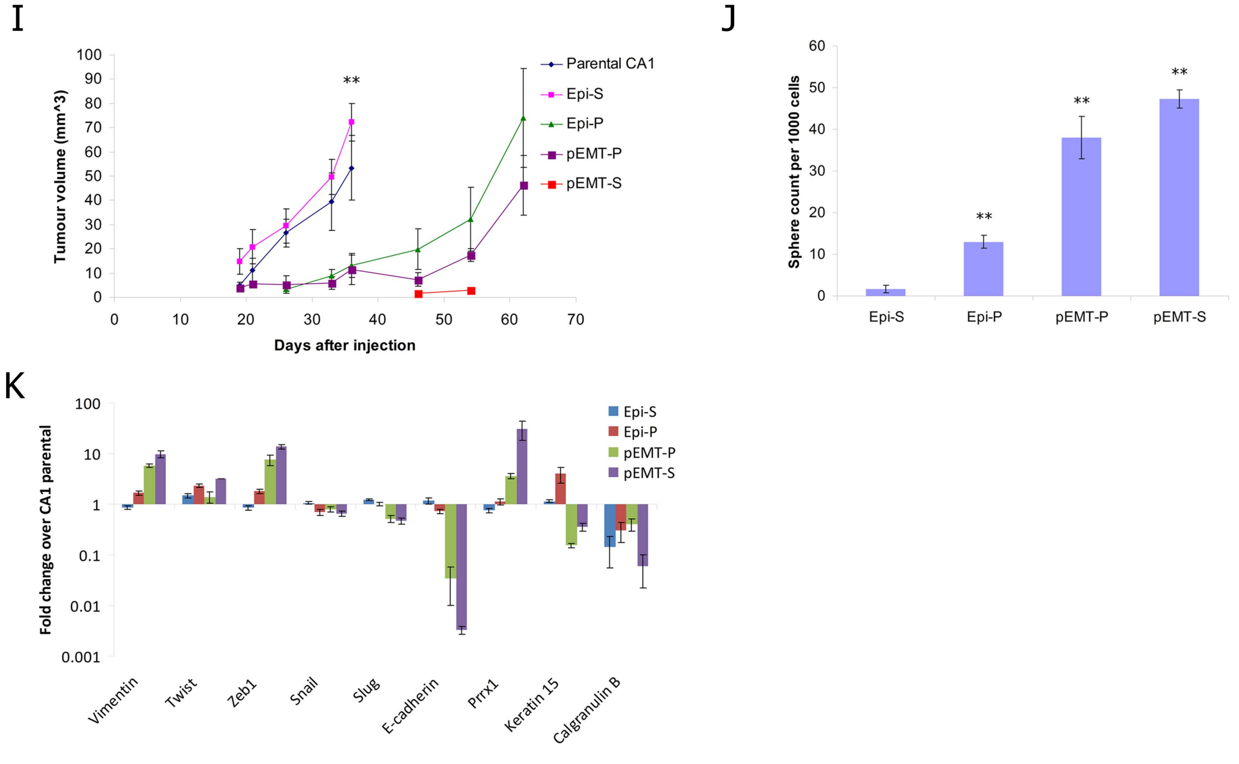

Figure S1 – Differences in epithelial-mesenchymal plasticity exist within both the epithelial and post-EMT CSC sub-populations, and relate to therapeutic resistance. A, Cell morphology of the parental CA1 cell line and four clonal sub-lines derived from this line. Epi-S; stable epithelial clonal sub-line. Epi-P; plastic epithelial clonal sub-line. pEMT-P; plastic post-EMT clonal sub-line. pEMT-S; stable post-EMT clonal sub-line. **B,** Size of the post-EMT sub-population ($CD44^{high}EPCAM^{low/-}$) as % of total cells in flow cytometric analysis under control conditions (left) or after 5 days treatment of the epithelial sub-lines with 5 ng/ml of the EMT-inducing cytokine TGFβ (right). These data demonstrate that Epi-P contains a larger post-EMT sub-population that Epi-S, and that EMT is stimulated by TGFβ to a much greater extent in Epi-P than in Epi-S. They also demonstrate that pEMT-P contains an epithelial sub-population, whereas pEMT-S does not. **C,** Assessment of plasticity over the first three passages of pEMT-P (left) and Epi-P (right) after plating early passage cell stocks. For pEMT-P, cells were re-plated for a further 7 days at each passage, followed by flow cytometric analysis to determine the size of the epithelial sub-population as a measure of the ability to undergo MET at each passage. For Epi-P, cells were treated for 5 days with 2 ng/ml TGFβ at each passage, followed by flow cytometric analysis to determine the size of the post-EMT sub-population and this reported as fold change compared to the baseline size of the post-EMT sub-population prior to administration of TGFβ. **D,** Cell morphology of pEMT-P (left) and Epi-P (right) over the first three passages after plating early passage cell stocks. At passages 2 and 3, cells with morphology similar to that of Epi-S can be seen appearing in Epi-P. **E,** IC50 values with 95% confidence intervals for the dose responses of each clonal sub-line and the parental CA1 cell line to cisplatin, paclitaxel and salinomycin. **F and G,** Assessment of CD24 staining over the first three passages of pEMT-P **(F)** and Epi-P **(G)** after

plating early passage cell stocks, with pEMT-S, Epi-S and the parental CA1 cell line for comparison. **H,** Tumour incidence for each sub-line upon injection of 5000 cells orthotopically into NOD/SCID mice. **I,** Growth curves for the tumours produced. **J,** Sphere count per 1000 cells plated into suspension culture, for each sub-line. Spheres appeared after 2-4 weeks culture. **K,** QPCR analysis of gene expression in each sub-line expressed as fold change compared to the parental CA1 line. For the panels in this figure, $n \geq 3$ biological repeats and error bars represent mean ± s.e.m. Significance (*$P<0.05$, **$P<0.01$) was determined by individual comparisons of the indicated treatment to each of the other treatments in **B** and **C**, by comparisons indicated by horizontal lines in **F** and **G**, by comparison of the parental CA1 line and Epi-S to Epi-P and pEMT-P in **I**, and by comparison of each of the other sub-lines to Epi-S in **J**.

Figure S2

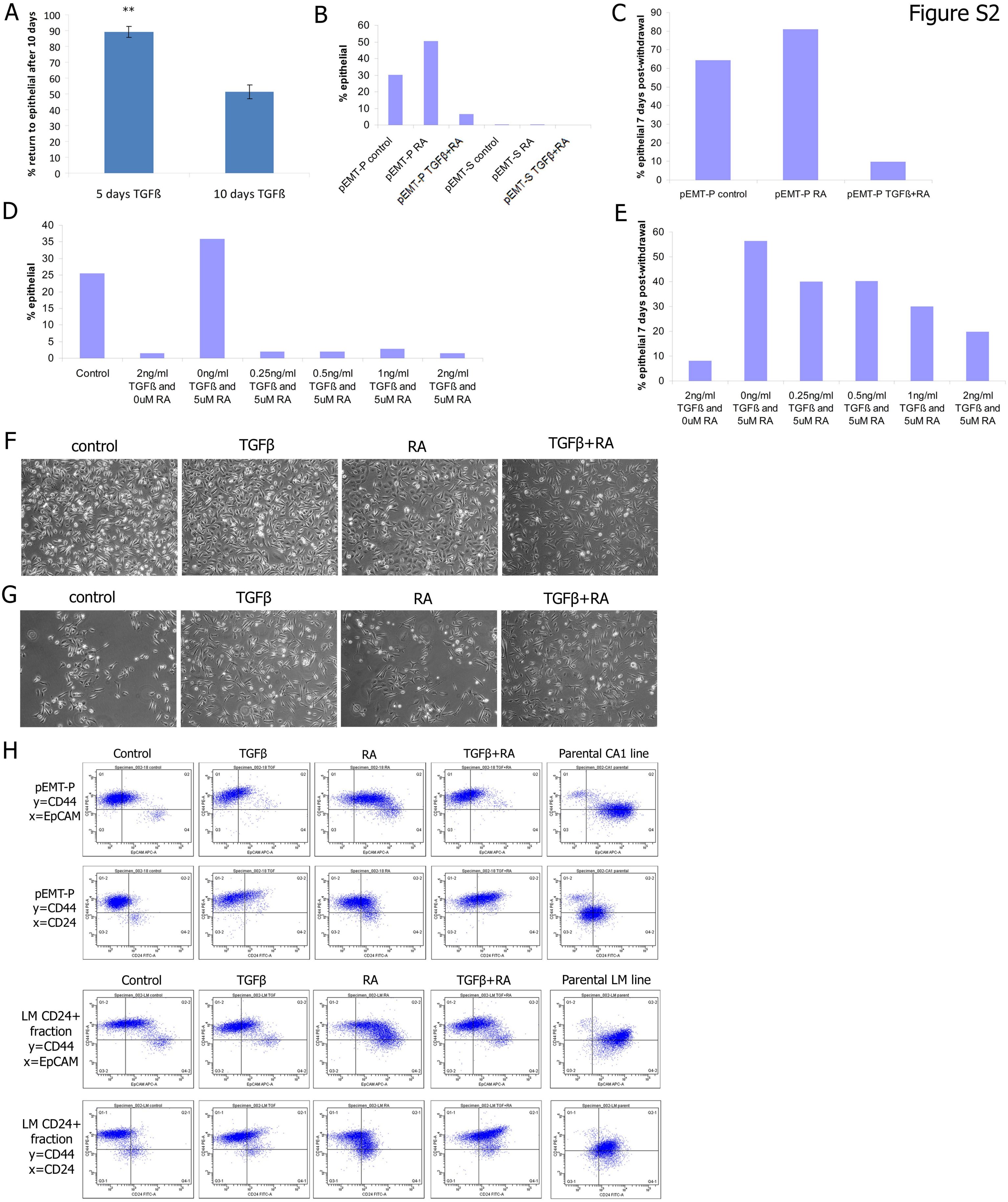

I

	Parental CA1	pEMT-P control	pEMT-P TGFβ	pEMT-P TGFβ+RA
Cisplatin IC50	0.3127	0.1931	0.0763	1.395
Cisplatin IC50 95% confidence intervals	0.2034 to 0.4808	0.1107 to 0.3370	0.02647 to 0.2200	1.075 to 1.812
Paclitaxel IC50	2.842	6.011	6.161	7.355
Paclitaxel IC50 95% confidence intervals	2.494 to 3.240	5.071 to 7.127	5.020 to 7.561	5.613 to 9.638

J

	Parental LM	CD24+ fraction control	CD24+ fraction TGFβ	CD24+ fraction TGFβ+RA
Cisplatin IC50	0.285	0.2513	0.4761	1.252
Cisplatin IC50 95% confidence intervals	0.1454 to 0.5588	0.1680 to 0.3759	0.3228 to 0.7022	1.040 to 1.507
Paclitaxel IC50	0.8363	2.345	1.706	1.557
Paclitaxel IC50 95% confidence intervals	0.4814 to 1.453	1.928 to 2.852	1.197 to 2.432	0.9845 to 2.463

Figure S2 – Combining TGFβ and RA blocks loss of plasticity and maintains therapeutic resistance. A, After treatment of the CA1 cell line with 5 ng/ml TGFβ for 5 or 10 days, the post-EMT fraction was FACS sorted and further cultured without TGFβ for 10 days, followed by flow cytometric analysis to determine the size of the epithelial sub-population (as % of total cells). Longer initial TGFβ treatment resulted in reduced ability to undergo MET. n = 3 and P<0.01. Error bars represent mean ± s.e.m. **B and C,** Flow cytometric analysis of pEMT-P and pEMT-S untreated (control) and after treatment with 5 uM RA (RA) or 5 ng/ml TGFβ and 5 uM RA (TGFβ+RA) (n = 1). The size of the epithelial sub-population **(B)**, and the size of the epithelial sub-population 7 days after withdrawal of the treatments from pEMT-P **(C)**, as % of total cells. **D and E,** Flow cytometric analysis of pEMT-P untreated (control) and after treatment with a range of doses of TGFβ with 5 uM RA (n = 1). The size of the epithelial sub-population **(D)**, and the size of the epithelial sub-population 7 days after withdrawal of the treatments from pEMT-P **(E)**, as % of total cells. **F and G,** Cell morphology of pEMT-P **(F)** and the $CD44^{high}EpCAM^{low/-}CD24^{+}$ fraction from the LM line **(G)** untreated (control) or treated with TGFβ, RA, or TGFβ+RA. **H,** Flow cytometry plots for pEMT-P and the $CD44^{high}EpCAM^{low/-}CD24^{+}$ fraction from the LM line, untreated (control) or treated with TGFβ, RA, or TGFβ+RA, and the corresponding parental line. The cell line and axis labels are indicated to the left of each set of plots, and the treatments are indicated above the plots. Crosshairs were set with isotype control. **I and J,** IC50 values with 95% confidence intervals for the dose response to cisplatin and paclitaxel of pEMT-P **(I)** and the $CD44^{high}EpCAM^{low/-}CD24^{+}$ fraction from the LM line **(J)** untreated (control) or treated with TGFβ or TGFβ+RA, and the corresponding parental line.

Figure S3

A

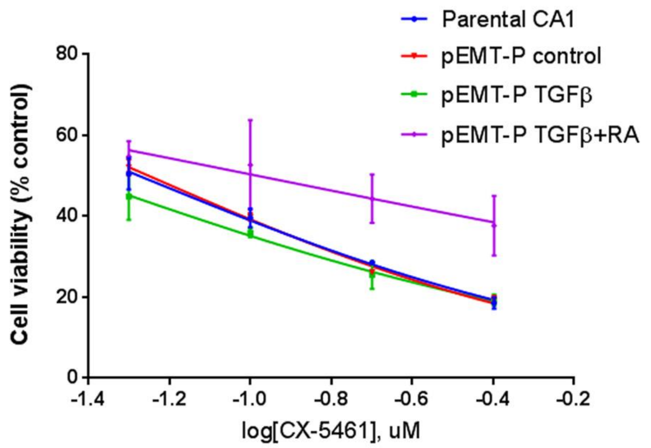

B

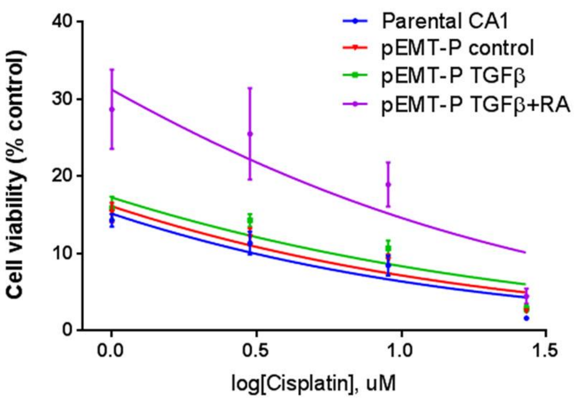

C

	Parental CA1	pEMT-P control	pEMT-P TGFβ	pEMT-P TGFβ+RA
CX-5461 IC50	0.05307	0.05606	0.03638	0.1044
CX-4561 IC50 95% confidence intervals	0.04225 to 0.06665	0.04990 to 0.06299	0.02191 to 0.06040	0.04041 to 0.2695
Cisplatin (+CX-5461) IC50	0.01622	0.01553	0.01271	0.155
Cisplatin (+CX-5461) IC50 95% confidence intervals	0.001662 to 0.1583	0.002115 to 0.1141	0.0009466 to 0.1706	0.01843 to 1.304

D

	Parental CA1	pEMT-P control	pEMT-P TGFβ	pEMT-P TGFβ+RA	pEMT-S
Bafilomycin IC50	0.327	0.2898	0.2606	0.285	0.4763
Bafilomycin IC50 95% confidence intervals	0.2288 to 0.4674	0.1880 to 0.4468	0.1547 to 0.4390	0.1627 to 0.4993	0.3550 to 0.6391
Tunicamycin IC50	0.004908	0.003447	0.004591	0.006477	0.002018
Tunicamycin IC50 95% confidence intervals	0.004434 to 0.005432	0.002869 to 0.004142	0.003751 to 0.005617	0.005421 to 0.007738	0.001585 to 0.002568
Thapsigargin IC50	0.003596	0.00318	0.0008084	0.001055	0.00126
Thapsigargin IC50 95% confidence intervals	0.002909 to 0.004445	0.002058 to 0.004912	0.0005082 to 0.001286	0.0009493 to 0.001172	0.0007393 to 0.002148

E

	Parental LM	CD24+ fraction control	CD24+ fraction TGFβ	CD24+ fraction TGFβ+RA
Bafilomycin IC50	0.4217	0.2066	0.2877	0.2778
Bafilomycin IC50 95% confidence intervals	0.3324 to 0.5350	0.1319 to 0.3236	0.2249 to 0.3680	0.1811 to 0.4262
Tunicamycin IC50	0.009029	0.001672	0.001624	0.001721
Tunicamycin IC50 95% confidence intervals	0.006440 to 0.01266	0.001317 to 0.002123	0.001355 to 0.001945	0.001450 to 0.002042
Thapsigargin IC50	0.002869	0.001391	0.001012	0.0005794
Thapsigargin IC50 95% confidence intervals	0.002138 to 0.003849	0.001075 to 0.001798	0.0008016 to 0.001277	0.0002652 to 0.001265

Figure S3 – Identifying compounds that target the drug resistant plastic post-EMT CSCs. A and B, Dose response to CX-5461 either alone at escalating doses **(A)** or at a fixed dose of 0.1 uM in combination with escalating doses of cisplatin **(B)** (n = 3 and error bars represent mean ± s.e.m). pEMT-P with no treatment (control) or treatment with TGFβ, RA, or TGFβ+RA, and the parental CA1 cell line. Cell viability expressed as number of cells remaining as a percentage of those in the control wells. **C,** IC50 values with 95% confidence intervals for the dose response to CX-5461 and cisplatin (+0.1 uM CX-5461) of pEMT-P untreated (control) or treated with TGFβ or TGFβ+RA, and the parental CA1 line. **D and E,** IC50 values with 95% confidence intervals for the dose response to bafilomycin A1, tunicamycin and thapsigargin of pEMT-P **(D)** and the $CD44^{high}EpCAM^{low/-}CD24^{+}$ fraction from the LM line **(E)** untreated (control) or treated with TGFβ or TGFβ+RA, and the corresponding parental line. Untreated pEMT-S was also included in **D**.

Figure S4

A

		Tumour number						
		1	2	3	4	5	6	7
Size of each fraction as % of total β4-integrin$^+$ tumour cells	CD44$^+$EpCAM^{high}CD24$^+$	5.2	13	1.7	1.6	5.3	14.8	22.5
	CD44$^+$EpCAM^{high}CD24$^-$	20.9	39.3	1.9	4.7	20.7	14	2.6
	CD44^{high}EpCAM$^{low/-}$CD24$^+$	1.3	0.29	16.7	0.8	1.2	0.81	1.6
	CD44^{high}EpCAM$^{low/-}$CD24$^-$	1.1	0.084	21.8	0.51	1.6	1.19	0.44

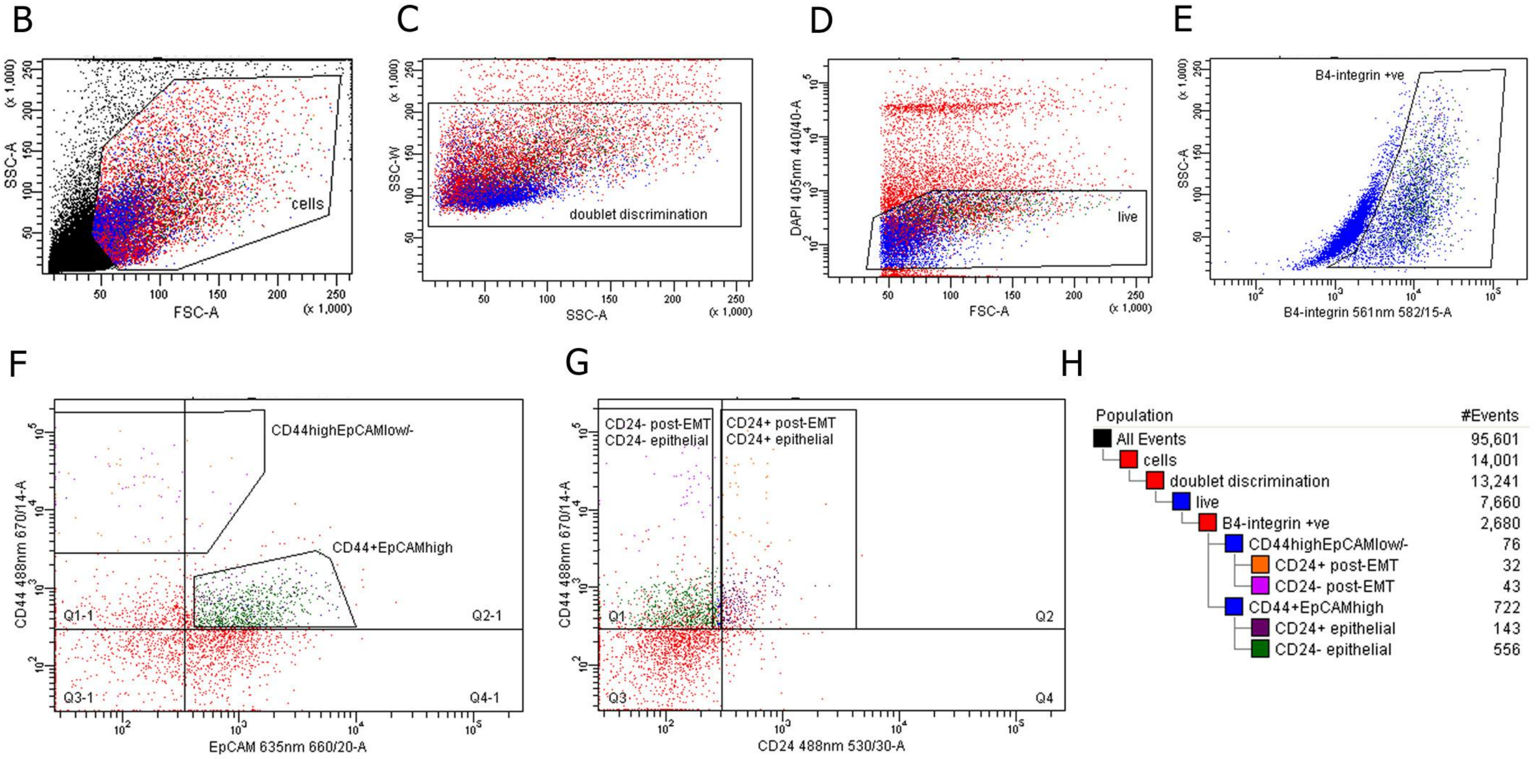

Figure S4 – Isolation of CSC sub-populations from fresh tissue specimens. A, Sizes of CSC sub-populations in flow cytometric analysis of 7 fresh tumour specimens. Overall cell distribution was similar across all tumour samples, but with occasionally marked differences in the sizes of the sub-populations. **B-G,** Representative FACS data for a tumour (the same tumour as in Figures 4A and 4B) showing the FACS gating strategy used to obtain the four CSC fractions. Sequential gating of cell-sized objects **(B)**, non-doublets **(C)**, live cells **(D)**, cells positive for the epithelial lineage marker β4-integrin used to separate tumour cells from non-tumour stromal cells **(E)**, $CD44^{high}EPCAM^{low/-}$ (post-EMT) and $CD44^{+}EpCAM^{high}$ (epithelial) sub-populations **(F)**, and CD24+/- epithelial and CD24+/- post-EMT sub-populations **(G)**. **H,** The population hierarchy used to obtain the four FACS sorted fractions ($CD24^{+}$ post-EMT, $CD24^{-}$ post-EMT, $CD24^{+}$ epithelial and $CD24^{-}$ epithelial), showing the number of cells in each fraction (#events) out of an analysis of 95601 total events (2680 β4-integrin^{+} tumour cells). β4-integrin stains tumour cells that have undergone EMT, but does not stain fibroblasts, so can separate tumour from stroma whilst retaining the post-EMT sub-population (L. Gammon, unpublished).

Table S1. Functional annotation clustering for genes upregulated in pEMT-P and Epi-P – see separate Excel file.

Table S2. Functional annotation clustering for genes upregulated in TGFβ and RA treated pEMT-P – see separate Excel file.

Table S3. Functional annotation clustering for genes upregulated in TGFβ and RA treated LM CD24+ fraction – see separate Excel file.

Supplementary Materials and Methods

Antibodies for Immunofluorescence

Primary antibodies were rabbit anti-pan-keratin (Abcam ab9377, used at 1:500 dilution), mouse anti-vimentin (clone V9, Dako, used at 1:500 dilution), rabbit anti-active caspase-3 (Millipore ab3623, used at 1:100 dilution) and mouse anti-LAMP-2 (clone H4B4, Biolegend, used at 1:100 dilution). Secondary antibodies were Alexa Fluor 488 goat anti-mouse (Life Technologies A-11001, used at 1:500 dilution) and Alexa Fluor 594 goat anti-rabbit (Life Technologies A-11037, used at 1:500 dilution).

Primer Sequences for QPCR

Primer sequences were as follows, all with a 60ºC annealing temperature; Vimentin F: CCCTCACCTGTGAAGTGGAT, Vimentin R: GACGAGCCATTTCCTCCTTC, Twist-1 F: GTCCGCAGTCTTACGAGGAG, Twist-1 R: CCAGCTTGAGGGTCTGAATC, Zeb1 F: GTCCAAGAACCACCCTTGAA, Zeb1 R: TTTTTGGGCGGTGTAGAATC, Snail F: CAAGGAATACCTCAGCCTGG, Snail R: CATCTGAGTGGGTCTGGAGG, Slug F: AGATGCATATTCGGACCCAC, Slug R: GCAGTGAGGGCAAGAAAAAG, E-cadherin F: GAACGCATTGCCACATACAC, E-cadherin R: AGCACCTTCCATGACAGACC, Prrx1 F: CTGATGCTTTTGTGCGAGAA, Prrx1 R: ACTTGGCTCTTCGGTTCTGA, Keratin 15 F: AGGTGTGCAGGCAGCTGTGTTTG, Keratin 15 R: AGAGGGTGTTGTGGGACCTCGT, Calgranulin B F: AAAGAGCTGGTGCGAAAAGA, Calgranulin B R: GTGTCCAGGTCCTCCATGAT.

Drugs

Cisplatin (Santa Cruz, SC-200896) was prepared as a 2 mM stock solution in 0.9% NaCl. Final concentrations were 1, 3, 9 and 27 uM. Paclitaxel (Sigma, T1912) was prepared as a 1 mM stock solution in DMSO. Final concentrations were 2.5, 5, 10 and 20 nM. Salinomycin (Sigma, S4526) was prepared as a 10 mM stock solution in DMSO. Final concentrations were 0.25, 1, 5 and 25 uM. CX-5461 (Caltag Medsystems, A11065) was prepared as a 10 mM stock solution in 50 mM monosodium phosphate. Final concentrations were 50, 100, 200 and 400 nM. Bafilomycin A1 (Sigma, B1793) was prepared as a 10 uM stock solution in DMSO. Final concentrations were 0.05, 0.2, 0.5 and 2 nM. Tunicamycin (Sigma, T7765) was prepared as a 1 mg/ml stock solution in DMSO. Final concentrations were 0.001, 0.003, 0.01 and 0.1 ug/ul. Thapsigargin (Sigma, T9033) was prepared as a 1 mM stock solution in DMSO. Final concentrations were 0.001, 0.01, 0.03 and 0.1 uM. All drug concentrations were determined empirically to give a dynamic range of effect.
